# Histological evidence for a supraspinous ligament in sauropod dinosaurs

**DOI:** 10.1098/rsos.150369

**Published:** 2015-10-28

**Authors:** Ignacio A. Cerda, Gabriel A. Casal, Rubén D. Martinez, Lucio M. Ibiricu

**Affiliations:** 1 CONICET-Instituto de Investigación en Paleobiología y Geología, Universidad Nacional de Río Negro, Museo Carlos Ameghino, Belgrano 1700, Paraje Pichi Ruca (predio Marabunta) 8300, Cipolletti, Río Negro, Argentina; 2 Laboratorio de Paleovertebrados, Universidad Nacional de la Patagonia San Juan Bosco, Ruta Prov. N° 1, Km 4, Comodoro Rivadavia (C.P. 9000), Chubut, Argentina; 3 CONICET-Centro Nacional Patagónico, Blvd. Alte. Brown 2915, Puerto Madryn, Argentina

**Keywords:** bone microstructure, functional morphology, Sauropoda, Titanosauria, metaplastic bone, mineralized fibres

## Abstract

Supraspinous ossified rods have been reported in the sacra of some derived sauropod dinosaurs. Although different hypotheses have been proposed to explain the origin of this structure, histological evidence has never been provided to support or reject any of them. In order to establish its origin, we analyse and characterize the microstructure of the supraspinous rod of two sauropod dinosaurs from the Upper Cretaceous of Argentina. The supraspinous ossified rod is almost entirely formed by dense Haversian bone. Remains of primary bone consist entirely of an avascular tissue composed of two types of fibre-like structures, which are coarse and longitudinally (parallel to the main axis of the element) oriented. These structures are differentiated on the basis of their optical properties under polarized light. Very thin fibrous strands are also observed in some regions. These small fibres are all oriented parallel to one another but perpendicular to the element main axis. Histological features of the primary bone tissue indicate that the sacral supraspinous rod corresponds to an ossified supraspinous ligament. The formation of this structure appears to have been a non-pathological metaplastic ossification, possibly induced by the continuous tensile forces applied to the element.

## 1. Introduction

**Figure 1.**
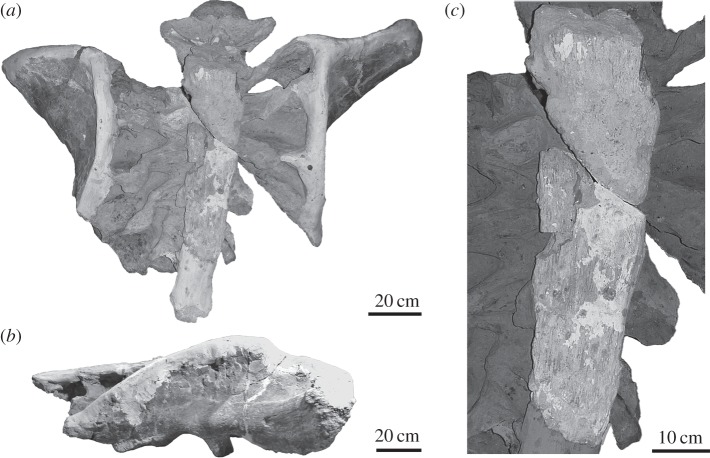
Sacrum of *Epachthosaurus sciuttoi* UNPSJB-PV 920 in dorsal (*a*) and right lateral (*b*) views and detailed view of the supraspinous rod over the sacral vertebrae spines (*c*). Sacral vertebrae and plaster reconstruction has been digitally obscured in (*c*).

Non-pathological intratendinous ossification is common in many dinosaurian clades [1]. This feature is particularly widespread in ornithischians, wherein it represents a synapomorphy of the clade [2,3]. Among theropod dinosaurs, ossified tendons in both the axial and appendicular skeleton are commonly formed in birds [4] and, to a minor degree, non-avian theropods [1,5]. With the exception of the hyperelongated cervical ribs of sauropods [6–8], evidence for intratendinous ossifications among sauropodomorph dinosaurs is rather scarce. One possible exception in this regard is the sacral supraspinous ossified rod reported in some derived taxa such as *Epachthosaurus sciuttoi* and *Malawisaurus dixei* [9–11]. This structure consists of an elongate, ossified rod that runs along the apices of the neural spines of the sacral vertebrae (figure 1). Although some authors have considered the supraspinous rod as an ossified tendon [12–14], others describe it as an ossified ligament [15,16] or even as a calcified cartilage [17]. Despite these different statements, no definitive evidence has been presented to support a tendinous, ligamentous or cartilaginous origin for this structure. This absence of information is rather noteworthy, because the determination of the possible tendinous, ligamentous or cartilaginous origin for the supraspinous rod has important (and very different in each instance) implications for the soft tissue reconstruction in sauropod dinosaurs.

Although palaeohistology has been demonstrated to be a valuable tool for the determination of the origin of different kinds of skeletal structures in fossil groups (i.e. [1,5,6,18–20]), no published study has provided histological evidence for the origin of the supraspinous ossified rod in sauropod dinosaurs. Giménez *et al*. [14] performed the only published histological study of this structure to date. They analysed thin sections from the supraspinous rod of *E. sciuttoi* from the Bajo Barreal Formation (Upper Cretaceous) of Argentina, which consist entirely of dense secondary (Haversian) bone tissue. Given that primary bone is the only tissue that can provide information about the histogenesis of the element, definitive evidence for the origin of the supraspinous rod cannot be obtained from the work of Giménez *et al*. [14].

In this study, we analyse and characterize the microstructure of the supraspinous rod of two sauropod dinosaurs from the Upper Cretaceous of Patagonia, Argentina. The two main goals of this work include (i) determining if the supraspinous rod derived from a tendinous, ligamentous or cartilaginous tissue and (ii) inferring the possible causes for the formation of this structure. We also evaluate the implications of the presence of a sacral supraspinous rod for soft tissue reconstruction in sauropod dinosaurs. Finally, as the sacral ossified rod has been considered an important taxonomic feature, we review the distribution of this character in sauropod dinosaurs. We considered that the histological study of the supraspinous rod in sauropod dinosaurs is important for three main reasons. First, the determination of its origin (from tendinous, ligamentous or cartilaginous structures) will improve our knowledge about those soft tissues related to the axial skeleton in sauropod dinosaurs, providing novel data for soft tissue reconstructions in this group. Second, the determination of the possible causes (e.g. pathological) for the supraspinous rod formation is fundamental to establish its validity as a character of taxonomic relevance. Finally, the microstructural characterization of the supraspinous rod will provide data about the possible functions of this structure.

### 1.1 Institutional abbreviations

MCS Ph, Museo de Cinco Saltos–Palaeohistological collection, Cinco Saltos, Argentina; MCS Pv: Museo de Cinco Saltos–palaeovertebrate collection, Cinco Saltos, Argentina; MCT Museu de Ciências da Terra of the Departamento Nacional de Produção Mineral, Rio de Janeiro, Brazil; MDT-Pv, Museo ‘Desiderio Torres’–Palaeovertebrate collection, Sarmiento, Chubut, Argentina; MPCA Ph, Museo Provincial ‘Carlos Ameghino’–Palaeohistological collection, Cipolletti, Argentina; MPCA Pv, Museo Provincial ‘Carlos Ameghino’–Palaeovertebrate collection, Cipolletti, Argentina; MUC, Museo de la Universidad Nacional del Comahue, Neuquén, Argentina; UNPSJB-PV, Universidad Nacional de la Patagonia ‘San Juan Bosco’–Palaeovertebrate collection, Comodoro Rivadavia, Argentina.

## 2. Material and methods

Sacral supraspinous ossified rods from *E. sciuttoi* and a still undescribed lithostrotian [11] (GA Casal, LM Ibiricu, RD Martinez, in preparation) were sampled for histological analysis. The material of *E. sciuttoi*(UNPSJB-PV 920) was collected from the locality of Estancia ‘Ocho Hermanos’, Sierra San Bernardo (Chubut Province, Patagonia, Argentina), in sediments that correspond with the upper portion of the Lower Member of the Bajo Barreal Formation (Upper Cretaceous: Late Cenomanian–Early Turonian) [9,21–23]. The indeterminate lithostrotian (MDT-Pv 4) consists of an incomplete but articulated skeleton collected in the locality of Rio Chico (Chubut Province, Patagonia, Argentina), from outcrops of Lago Colhué Huapi Formation (Coniacian–Maastrichtian) [24]. The presence of osteoderms and the procoelous condition of the anterior caudal vertebra allows its inclusion within Lithostrotia [25,26]. The anatomical description and precise systematic affinities of this specimen will be published elsewhere (GA Casal, LM Ibiricu, RD Martinez, in preparation). The samples obtained for thin sectioning were taken from the caudal-most portion of the supraspinous rod in both specimens.

Given that previously invoked hypotheses propose that the supraspinous rod originated from the mineralization of a cartilaginous [17], tendinous [12–14] or ligamentous tissue [15,16], we compare the histology of these particular tissues with the data obtained from *E. sciuttoi*and MDT-Pv 4. For this, we examine thin sections of ossified tendons, and calcified cartilage (hyaline and fibrous) obtained from different dinosaur taxa from Argentina. Data on section planes, accession numbers, localities and horizons of these samples are compiled in table 1. The studied ossified tendons correspond with hypertrophied cervical ribs of titanosaurian sauropods and caudal ossified tendons of the basal ornithopod dinosaur *Gasparinisaura cincosaltensis*. The ossified tendons (cervical ribs) of an indeterminate titanosaur and *Bonitasaura salgadoi* were previously studied by Cerda [6] and Gallina [7], respectively. The fibrous cartilage was studied from the articular surface of a single prezygapophysis obtained from a mid-caudal vertebra of an indeterminate lithostrotian titanosaur [27]. Samples of hyaline calcified cartilage were examined from the articular surfaces of appendicular bones of *G. cincosaltensis* and from an undetermined titanosaur (table 1). We complement our first hand observations with data obtained from several published studies on both mineralized and unmineralized tendinous, ligamentous and cartilaginous tissues.

**Table 1.** Elements sectioned for this study.

taxon	element	specimen number	slide number	provenance	horizon	age
*Epachthosaurus sciuttoi*	sacral supraspinous rod	UNPSJB-PV 920	UNPSJB-PV 920/1, 920/2, 920/3, 920/4	Sierra de San Bernardo, Chubut Province	Bajo Barreal Formation	Late Cenomanian–Early Turonian
*Epachthosaurus sciuttoi*	femur	UNPSJB-PV 920	UNPSJB-PV 920/5, 920/6, 920/7	Sierra de San Bernardo, Chubut Province	Bajo Barreal Formation	Late Cenomanian–Early Turonian
Lithostrotia indet.	sacral supraspinous rod	MDT-Pv 4	MDT-Pv 4/1, 4/2, 4/3, 4/4, 4/5	Rio Chico, Chubut Province	Lago Colhué Huapi Formation	Coniacian–Maastrichtian
*Bonitasaura salgadoi*	cervical rib	MPCA Pv 460	MPCA Ph 460/9, 460/10	Cerro Policía, Río Negro Province	Bajo de la Carpa Formation	Santonian
Titanosauria indet.	cervical rib	MPCA Pv uncatalogued	MUC Ph 139, 140, 141, 142, 138	El Anfiteatro, Río Negro Province	Plottier Formation	Late Coniacian
Titanosauria indet.	cervical rib	MCS Pv uncatalogued	MCS Ph 78	Cinco Saltos, Río Negro Province	Anacleto Formation	Early Campanian
*Gasparinisaura cincosaltensis*	caudal ossified tendons	MCS Pv 112	MCS Ph 26, 27, 28, 29, 30.	Cinco Saltos, Río Negro Province	Anacleto Formation	Early Campanian
*Gasparinisaura cincosaltensis*	femur (proximal end)	MCS Pv 3	MCS Ph 6	Cinco Saltos, Río Negro Province	Anacleto Formation	Early Campanian
*Gasparinisaura cincosaltensis*	tibia (proximal end)	MCS Pv 2	MCS Ph 7	Cinco Saltos, Río Negro Province	Anacleto Formation	Early Campanian
Titanosauria indet.	metatarsal (proximal end)	MCS Pv 174/11	MCS Ph 77	Cinco Saltos, Río Negro Province	Anacleto Formation	Early Campanian
Lithostrotia indet.	caudal vertebra (prezygapophysis)	MCS Pv 183/3	MCS Ph 76	Cinco Saltos, Río Negro Province	Anacleto Formation	Early Campanian

Specimens were prepared for thin sections based on the methodology outlined in Chinsamy & Raath [28]. The preparation of the histological sections was carried out in the Departamento de Geología de la Universidad Nacional de San Luis (Argentina). The slices were studied using a petrographic polarizing microscope (Nikon E200 pol). Nomenclature and definitions of structures used in this study are derived from Francillon-Vieillot *et al*. [29] and Chinsamy-Turan [30].

## 3. Description

### 3.1 Supraspinous rod

#### 3.1.1 Lithostrotia indet. MDT-Pv 4

**Figure 2.**
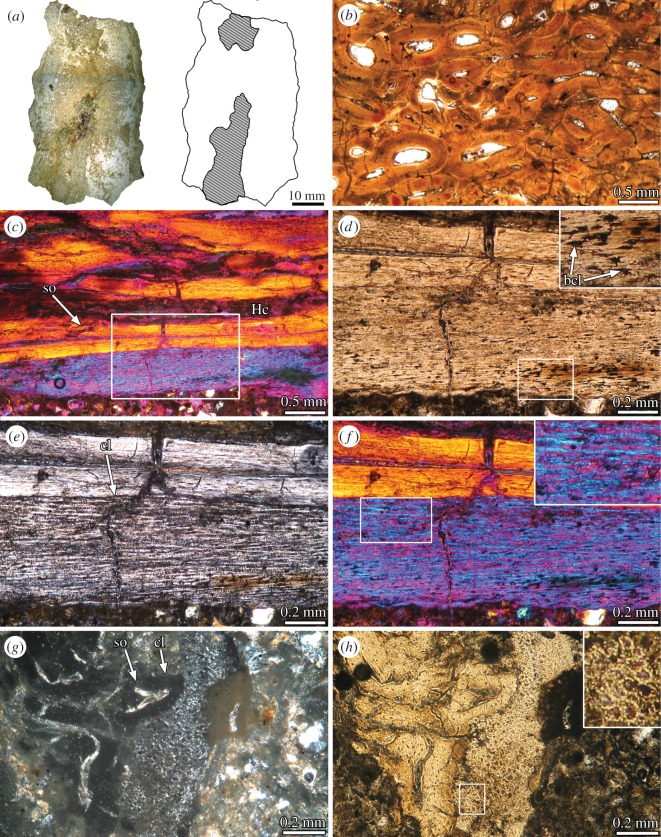
Bone histology of the sacral supraspinous rod of Lithostrotia indet. MDT-Pv 4. (*a*) Complete cross section of the element. The areas occupied by bone tissue (white) and sediment (dashed lines) are shown in the schematic drawing. (*b*) Dense Haversian tissue in transverse section. (*c*) Dense Haversian bone and unremodelled primary bone in longitudinal section. (*d*–*f*) Close-up of the same region (box inset in (*c*)). Detailed view of the bone cell lacunae are shown in the upper right corner in (*d*). Detailed view of the fibres are showed in the upper right corner in (*f*). Note the strong variation in the optical properties between the primary and secondary bone. (*g*,*h*) Detailed view of unremodelled primary bone tissue in transverse section. Note the irregular shape of the transversely sectioned fibres. (*a*,*b*,*d*,*h*) normal light; (*e*,*g*) cross-polarized light; (*c*,*f*) cross-polarized light with lambda compensator. bcl, bone cell lacunae; cl, cementing line; Hc, Haversian canal; so, secondary osteon.

In transverse cross section, the element possesses a narrow profile, that is much higher than wide. To the naked eye, the structure appears to possess an internal ‘cavity’, which is open ventrally in the medial region and extends far toward the dorsal edge (figure 2*a*). However, based on the histological data, this ‘cavity’ appears to be a space between two fragments of the osseous structure, which was broken in two halves. If our interpretation is correct, the osseous element is strongly dorsoventrally flattened.

Unfortunately, the sample has undergone very important diagenetic alteration, which makes it difficult to properly describe and interpret the bone tissues. The following description is based on the best-preserved areas, which still maintain their main histological features. The structure is composed entirely by compact bone, which is mostly formed by dense Haversian tissue (figure 2*b*). Partly overlapping secondary osteons of different generations and in different stages of development are profusely distributed in the compacta. These osteons are formed by centripetally deposited lamellar bone and they show an important degree of variation with regard to their diameter. Although Haversian canals are mainly oriented parallel to the element main axis, variation does occur. In several areas (interpreted here as the ventral region), large secondary osteons exhibit variable orientation (e.g. perpendicular to the sagittal plane). In these areas also there is commonly the abundant presence of interstitial lamellar bone, which appears to correspond to remains of prominent internal cavities.

Despite the profuse secondary remodelling observed in the sample, remains of primary bone tissue are preserved in some regions. These areas correspond to the outermost external cortex and some areas that surround the ‘internal cavity/cavities’ (ventral cortex?). Primary bone is best observed in longitudinal sections. This tissue is avascular and exhibits a coarsely fibrous-like texture (figure 2*c*–*f*). Bone cell lacunae are elongated in shape, which differs from the strongly flattened appearance of the osteocyte lacunae of the secondary bone. The extracellular matrix is composed of abundant mineralized collagenous fibres that run parallel or sub-parallel to the element main axis (figure 2*e*,*f*). Under polarized light, these fibres are birefringent in longitudinal section. The collagenous fibres are intercalated with other structures, which also possess a fibrilar appearance but differ in their optical properties (monorefringent under polarized light). As with the collagenous fibres, these structures are coarse and they run parallel to the element main axis. The presence of both types of fibrilar structures regularly intercalated in the matrix give the bone tissue a ‘striated’ pattern of birefringence under polarized light. This pattern contrasts with the homogeneous and strongly birefringent appearance of the secondary lamellar bone of the surrounding Haversian systems (figure 2*e*,*f*). Very thin fibrous strands are observed in some regions and only using high magnifications (e.g. 400×). When they are present, these small fibres are oriented parallel to each other but perpendicular to the rod main axis. Their density is important in some regions. In transversal sections, the osseous matrix exhibits a mass monorefringence, which is interrupted by thin and short ‘micro-patches’ of birefringent tissue (figure 2*g*). In the monorefringent portion of the matrix, several dark coloured structures of rounded or irregular contours are observed embedded in a brighter matrix (figure 2*h*).

#### 3.1.2 *Epachthosaurus sciuttoi* UNPSJB-Pv 920

**Figure 3.**
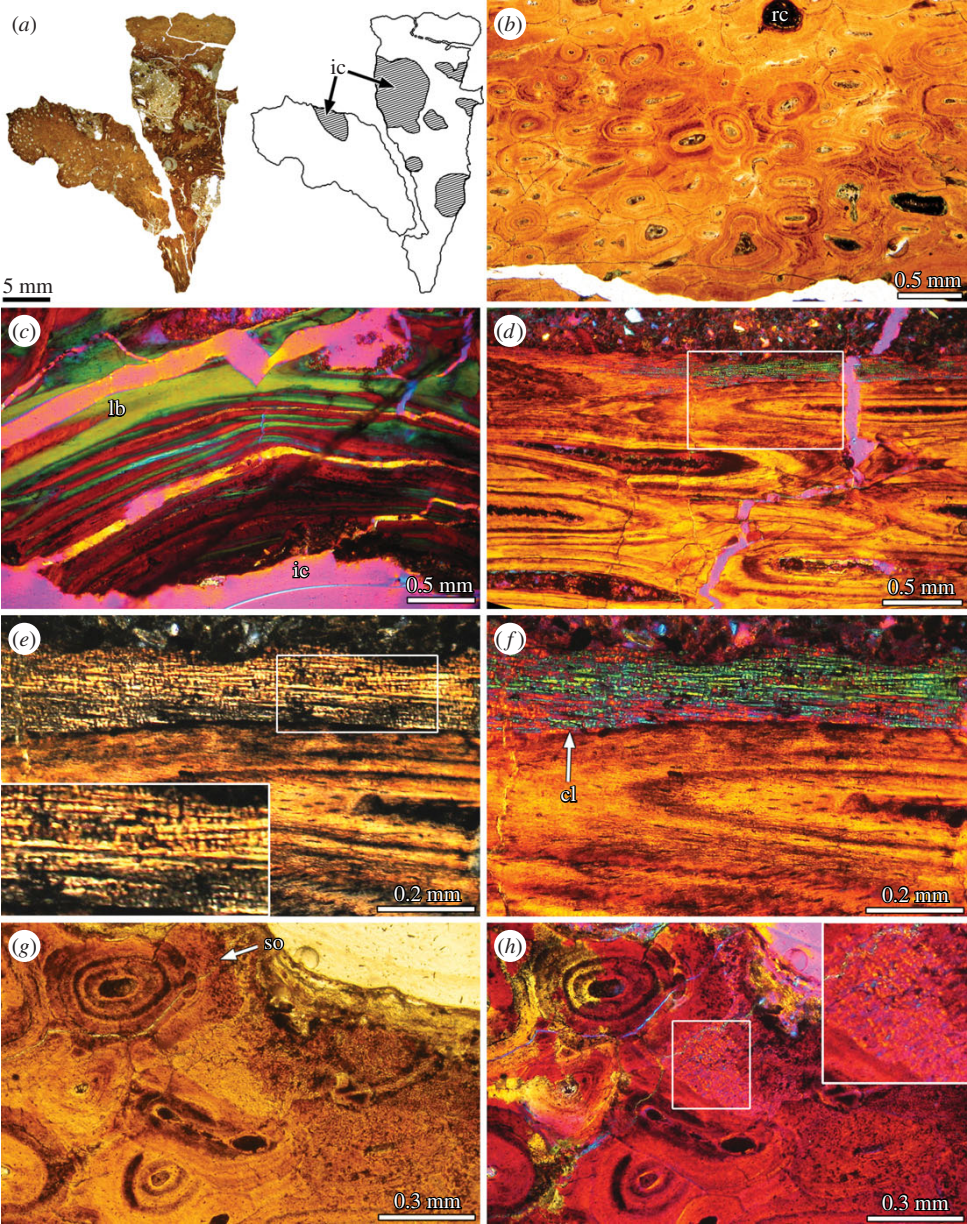
Bone histology of the sacral supraspinous rod of *Epachthosaurus sciuttoi* UNPSJB-PV 920. (*a*) Complete cross section of the element. The areas occupied by bone tissue (white) and sediment (dashed lines) are showed in the schematic drawing. The areas filled with sediment correspond with large, irregular internal spaces. (*b*) Dense Haversian tissue in transverse section. (*c*) Detailed view of the lamellar bone tissue formed around one of the large internal cavities. Note the strong variation in the fibre orientation between successive lamellae, evidenced by the optical properties of the secondary bone. (*d*) Dense Haversian bone and unremodelled primary bone in longitudinal section. (*e*,*f*) Close-up of the same region (box inset in (*d*)). Detailed view of the fibres are showed in the lower left corner in (*e*). Note the presence of thin, transversally oriented fibres (arrowheads). (*g*,*h*) Detailed view of secondary osteons and unremodelled primary bone tissue in transverse section. Detailed view of the primary bone is showed in the upper right corner in (*g*). (*a*,*b*,*g*) Normal light; (*e*) cross-polarized light; (*c*,*d*,*f*,*h*) cross-polarized light with lambda compensator. cl, cementing line; ic, internal cavity; lb, lamellar bone; so, secondary osteon.

The obtained sample is composed of compact bone tissue, which exhibits a striated or ‘fibrous’ aspect even in the broken surfaces. The internal portion reveals the presence of very large (approx. 1–8 mm in diameter) canals, which possess irregular shapes (figure 3*a*). The broken surfaces show that these cavities are vermiform rather than straight and that they anastomose in different directions.

Despite the important degree of diagenetic alteration observed in several portions of the sample, most of the tissue exhibits good histological preservation. The compact bone shows extensive processes of secondary remodelling, resulting in a dense Haversian bone which tends to obliterate almost entirely the primary tissues (figure 3*b*). Resorption cavities and secondary osteons of different generations and in different stages of development are mostly oriented parallel to the structure major axis. Laterally, larger resorption cavities tend to coalesce and form larger irregular spaces. In this area, resorption cavities and Haversian osteons in early stages of development are more commonly observed than in other regions. The large internal cavities are lined by thick layers of lamellar bone tissue (figure 3*c*). The resorption line that marks the beginning of the centripetal bone deposition around the large cavities interrupts several neighbouring secondary osteons. Also, several resorption cavities and secondary osteons disrupt the lamellar bone that lines the internal cavities.

Primary bone tissue has only been preserved in very small portions of the sample. This tissue exhibits the same features observed in MDT-Pv 4. In this regard, the primary bone tissue is avascular and shows a coarsely fibrous texture (figure 3*d*). The matrix is formed by the two main types of coarse, longitudinally oriented fibres (mono- and birefringent under polarized light in longitudinal sections; figure 3*e*,*f*). In contrast with the structure observed in MDT-Pv 4, in which both kinds of fibrilar structures were regularly intercalated, some areas of the *E. sciuttoi* sample exhibit greater density of one or other type of fibre. The fine striation observed in MDT-Pv 4 is also present in *E. sciuttoi*. Such striation is perpendicular to the main coarse fibres and is better discerned under polarized light (figure 3*e*). Very small patches of primary bone are observed in transverse sections. As observed in MDT-Pv 4, the osseous matrix is avascular, mostly monorefringent and with thin and short patches of fibrous strands.

### 3.2 Ossified tendons

#### 3.2.1 Titanosaurian cervical ribs

**Figure 4.**
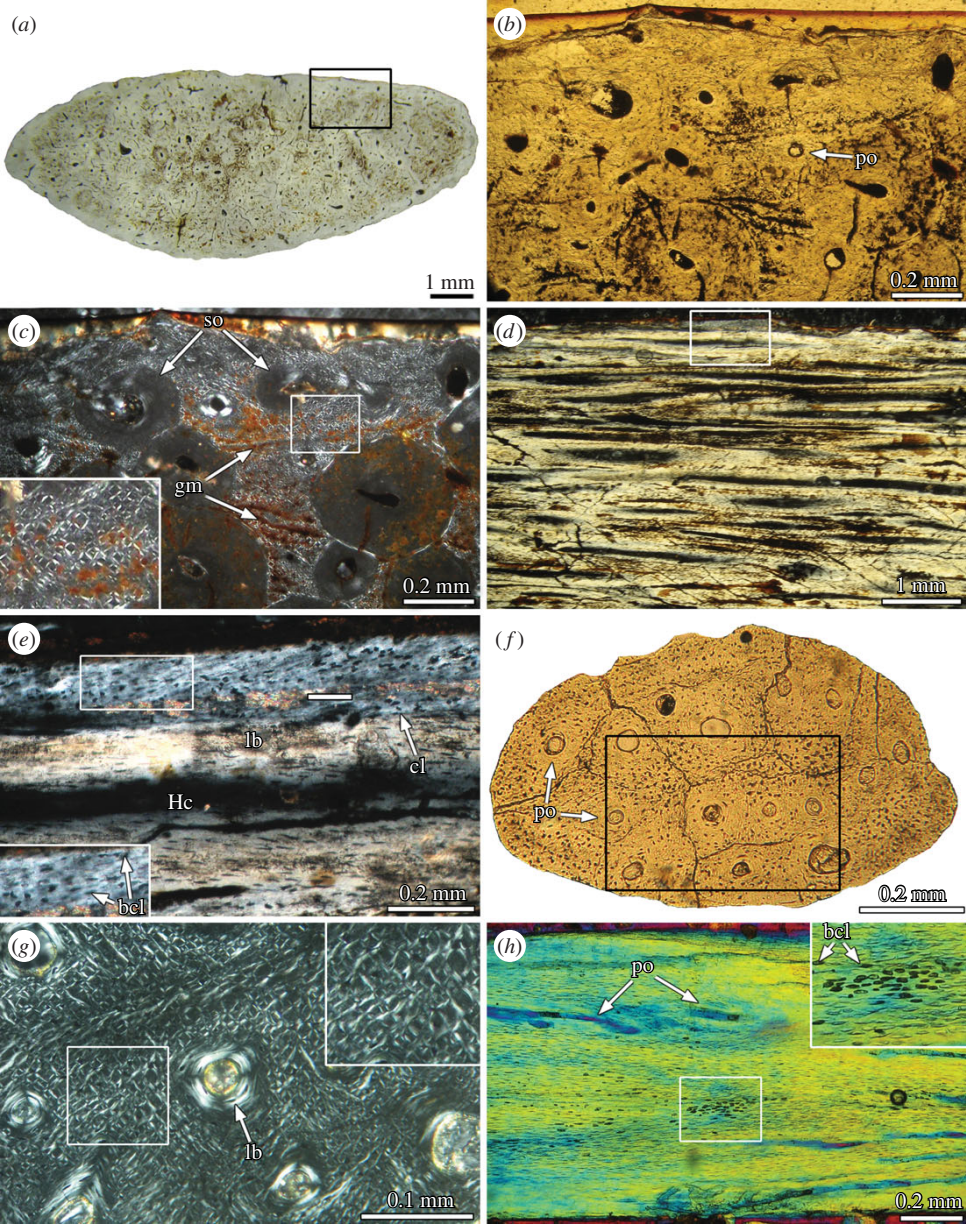
Cervical rib histology of Titanosauria indet. MCS Pv uncatalogued specimen (*a*–*e*) and ossified tendons of *Gasparinisaura cincosaltensis* MCS Pv 112 (*f*–*h*). (*a*) Complete cross section of the element. (*b*,*c*) Detailed view of secondary osteons and unremodelled primary bone tissue in transverse section (box inset in (*a*)). Detailed view of the fibres are showed in the lower left corner in (*c*). Note the fine white lines delineating each single fibre and the rhomboidal shape of these. (*d*) General view of the dense Haversian bone in longitudinal section. (*e*) Close-up of the unremodelled primary bone at the outer cortex (box inset in (*d*)). Detailed view of the bone cell lacunae are showed in the lower left corner in (*e*). (*f*) Complete cross section of the element. (*g*) Close-up of the primary bone tissue. Detailed view of the fibres is showed in the upper right corner. Compare the appearance of the transversally sectioned fibres with (*c*). (*h*) Longitudinally sectioned ossified tendon. Detailed view of the bone cell lacunae is showed in the upper right corner. (*a*,*b*,*f*) Normal light; (*c*–*e*,*g*) cross-polarized light; (*h*) cross-polarized light with lambda compensator. bcl, bone cell lacunae; cl, cementing line; gm, growth mark; Hc, Haversian canal; lb, lamellar bone; po, primary osteon; so, secondary osteon.

The samples from *Bonitasaura* and the two indetermined titanosaurs show similar microstructures; for this reason, they will be described together. According to the previously published descriptions of Cerda [6] and Gallina [7] for the same samples, the cervical ribs are formed entirely by compact bone, which mostly correspond with dense Haversian bone (figure 4*a*). Several superimposed generations of secondary osteons are longitudinally oriented (figure 4*b*–*d*). Volkmann canals occasionally connect the Haversian systems. The sections reveal that the bone remodelling was in progress at the moment of the death of the individuals, because some resorption cavities and many immature secondary osteons are present throughout the compacta. Primary bone tissue is preserved in the outermost region of the cortex (figure 4*d*). The major proportion of primary bone is observed toward the distal end of the rib. According to previous descriptions, the primary bone is formed by coarse bundles of mineralized collagenous fibres (figure 4*e*). These fibres are oriented parallel to the rib main axis. In transverse sections, the mineralized extracellular matrix is monorefringent and the delineations of individual fibres are visible as thin bright lines. Conversely, the primary matrix exhibits a mass birefringence in longitudinal sections. This birefringence is, however, not as strong as the lamellar bone tissue of the adjacent secondary osteons (figure 4*e*). In this sense, the primary bone exhibits the typical appearance of the parallel fibred bone, in which the degree of organization of the intrinsic collagenous fibres is lesser than in the lamellar bone [29]. Bone cell lacunae are elongated and they follow the fibre orientation. Few primary osteons (‘secondary reconstructions’ *sensu* Horner *et al.* [5]) are embedded in the matrix. At least two growth marks can be discerned in the primary bone of one of the indetermined titanosaurs (figure 4*c*).

#### 3.2.2 *Gasparinisaura* ossified tendons

These thin and long elements are entirely formed by compact primary bone tissue (figure 4*f*). As described in the titanosaur cervical ribs, the matrix is composed of coarse mineralized fibres oriented parallel to the tendon longitudinal axis (figure 4*g*). Bone cell lacunae are slightly or pronouncedly elongated and they are aligned with the intrinsic fibres. They are commonly abundant and form large clusters in some areas, mostly at the inner region (figure 4*h*). Vascularization consists of longitudinally oriented canals lined by lamellar bone (primary osteons). In longitudinal sections, the degree of birefringence is more pronounced around the vascular spaces than in the fibrous matrix. No growth marks were observed.

### 3.3 Calcified cartilage

#### 3.3.1 Metatarsal articular surfaces

**Figure 5.**
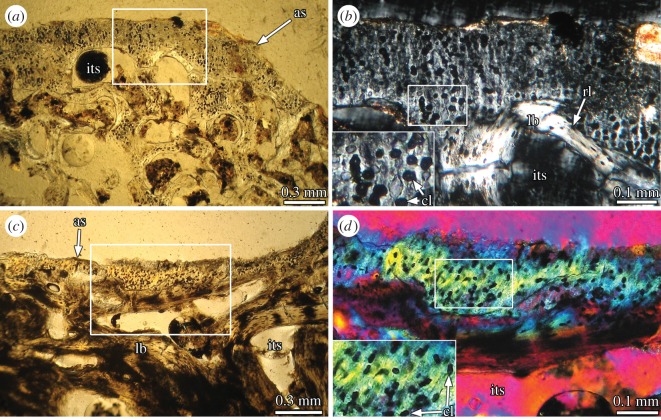
Calcified cartilage of Titanosauria indet. MCS-Pv 174/11 (*a*,*b*) and Lithostrotia indet. MCS-Pv 183/3 (*c*,*d*). (*a*) longitudinal section of a metatarsal proximal end. (*b*) Detail of the same section (box inset in (*a*)) showing the calcified hyaline cartilage. (*c*) Transverse section of a prezygapophysis. (*d*) Detail of the same section (box inset in (*c*)) showing the calcified fibro cartilage. (*a*,*c*) Normal light; (*b*) cross-polarized light; (*d*) cross-polarized light with lambda compensator. as, articular surface; cl, condrocyte lacunae; its, intertrabecular space; lb, lamellar bone; rl, resorption line.

Longitudinal sections of long bones are composed of coarse cancellous bone and a thin layer of calcified hyaline cartilage (figure 5*a*). The cancellous bone is formed by thin trabeculae of secondary lamellar bone tissue, with characteristically flattened osteocyte lacunae immersed in a strongly birefringent matrix. Cementing lines separate different generations of continuous erosion and formation of lamellar bone. Calcified cartilage consists of abundant hypertrophic chondrocytic lacunae of globular shape (figure 5*b*). Only a thin layer of calcified matrix separates each lacuna. No traces of fibrous tissue are recognized in this matrix. The arrangement of the chondrocytic lacunae varies between being organized in vertical columns (titanosaurs) and highly disorganized (*Gasparinisaura*).

#### 3.3.2 Prezygapophyseal articular surface

The internal structure of the prezygapophysis is mainly composed of dense Haversian bone tissue. Secondary osteons are abundant and reach the outermost portion of the cortex. Several of them exhibit wide vascular spaces, indicating an active remodelling process at the moment of the death. Although Haversian canals are mainly longitudinally oriented, they are aligned almost perpendicularly to the outer cortex toward the articular surface.

The primary bone tissue is restricted to a thin layer (50–700 μm) located at the outermost cortex. Except for the articular region, the primary bone corresponds with a parallel fibred bone tissue. At the articulation region, a very thin layer of calcified fibrocartilage is observed (figure 5*c*). This tissue is avascular and consists of abundant and fine fibres aligned in parallel that runs perpendicularly to the surface. Chondrocyte lacunae are mostly rounded (figure 5*d*). In several regions, these lacunae are roughly aligned in columns that follow the fibre orientation. These observations coincide with previous descriptions of calcified fibrocartilage [31,32].

## 4. Discussion

### 4.1 Tendon, ligament or cartilage? The origin of the supraspinous rod

As the microstructure of the primary bone tissues can provide information about the origin of singular skeletal structures, it is possible to test the different hypotheses proposed for the formation of the supraspinous rod in sauropod dinosaurs. Previously invoked hypotheses propose that the supraspinous rod originated from the mineralization of cartilaginous [17], tendinous [12–14] or ligamentous tissue [15,16]. If the supraspinous rod originated from a cartilaginous structure, we would expect to find remains of calcified hyaline or fibrous cartilage in the non-remodelled areas of the element. The microstructure of the primary bone tissue of the supraspinous rod in *E. sciuttoi* and MDT-Pv 4 (figures 2*f* and 3*e*) is characterized by the presence of at least three different types of fibre-like structures. These structures are differentiated on the basis of relative size, spatial orientation and optical properties under polarized light. This complex structure differs from those observed in the hyaline and fibrous calcified cartilage in our sample (figure 5*b*,*d*) and in other previous studies (e.g. [31–35]). The mineralized extracellular matrix of the calcified hyaline cartilage is relatively sparse, with a relatively low content of fibres. This tissue is also characterized by the abundance of globose chondrocyte lacunae. With respect to the mineralized fibrocartilage, although this tissue is rich in collagenous fibres, these are fine and oriented in a single direction (birefringent when longitudinally sectioned). Such microstructure is roughly similar to that observed in ossified tendons (see below). The absence of histological features related to calcified cartilage does not support the ‘cartilaginous’ hypothesis for the supraspinous rod in our sample. In a recent contribution, Horner *et al*. [5] described a particular bone tissue in the interspinous ligament scars from *Diplodocus*, which was interpreted as the calcified fibrocartilage of the interspinous ligament enthesis. Although the presence of thin ‘tube like’ structures described in this tissue resembles the coarse monorefringent fibre-like structure from the supraspinous rod in *E. sciuttoi* and MDT-Pv 4, the absence of fine, transversely oriented fibres in the interspinous scar indicates that the nature of this structure is actually different from the supraspinous rod. Furthermore, given that fibrocartilage is commonly observed in ‘transition’ areas (e.g. neurocentral sutures, tendon and ligament entheses), it appears to be improbable that a distinctive and well differentiated structure as the supraspinous rod originates entirely from the calcification of a fibrocartilaginous element. The absence of cartilaginous precursors indicates that the supraspinous rod did not originate from a typical endochondral ossification.

Regarding the ‘tendinous’ and ‘ligamentous’ hypotheses, both ideas imply the transformation into bone via metaplasia (i.e. ossification of a fully differentiated, non-osseous tissue, without the involvement of true osteoblasts) of a tendinous or ligamentous structure (evolutionary definition of metaplasia *sensu* Horner *et al.* [5]). When metaplastic ossification occurs, the primary bone tissue commonly exhibits the histological features from the original, non-osseous tissue (i.e. ligament or tendon) from which the osseous element (i.e. supraspinous rod) originated. In the case of tendons, these structures are mainly formed by coarse collagenous fibre bundles oriented parallel to the element main axis. Fibroblasts (tenocytes) are elongate and run alongside the collagenous fibres ([1,36–39], but see [5] for a different interpretation). During intratendinous ossification, fibroblasts proliferate and hypertrophy, and vascularization increases [36]. These histological features are maintained in the primary bone tissue and they are clearly observed in the ossified tendons of *Gasparinisaura* and other ornithischians ([1,39–41], this work) and in the cervical ribs of sauropod dinosaurs ([6–8,42], this work). The rather simple microstructure of the ossified tendons departs from the more complex structure observed in the primary bone tissue of the supraspinous rod.

In the case of ligamentous structures, the microstructure of the sauropod supraspinous rod can be compared with two main types of ligament, which are mainly differentiated on the basis of their relative composition of collagenous and elastic fibres (collagenous and elastic ligaments). The gross histological features of collagenous ligaments (e.g. cruciate ligament) do not appear to exhibit important variations in comparison to tendons [37,43–47]. In this sense, the histology of collagenous ligaments exhibits a rather simple structure, in which the matrix is poorly vascularized and coarse mineralized fibres oriented in parallel predominate [37,43–47]. Again, the histological features of the supraspinous rod in sauropods differ from those reported for collagenous ligaments.

Regarding elastic ligaments (e.g. nuchal ligament), these structures are formed by both collagenous and elastic fibres [47–55]. In a detailed study of the bovine nuchal ligament, Morocutti *et al*. [50] showed that this element consists of longitudinally oriented elastic and collagenous fibres and very thin collagenous fibres transversely oriented. Elastic fibres are coarse (20 μm diameter) and they are embedded in a matrix of collagenous fibres. The microstructure of the sauropod supraspinous rod strongly resembles that reported in different types of elastic ligaments (e.g. [47,49,50,52,53,55,56]). We suggest that the birefringent fibres observed in longitudinal sections in the sauropod supraspinous rod actually correspond to the matrix of longitudinally oriented collagenous fibres, which are mineralized in the supraspinous rod. In the same way, the coarse, monorefringent fibrous structures correspond to the elastic fibres of the ligament. The monorefringent nature of these fibres is possibly related to the amorphous structure of the mineralized elastic proteins [57]. Finally, we consider that the fine fibrous-like structures oriented perpendicularly to the rod main axis correspond to the fine collagenous fibres, similar to those described in the nuchal ligament [50]. Hence, in line with previously proposed hypotheses for the origin of the supraspinous rod in sauropod dinosaurs, the matrix of the primary bone tissue of the supraspinous rod of *E. sciuttoi* and MDT-Pv 4 specimen supports a ligamentous origin for this structure. In particular, our comparative analysis strongly suggests that the supraspinous rod corresponds to a mineralized elastic ligament.

### 4.2 Causes for ligament ossification

**Figure 6.**
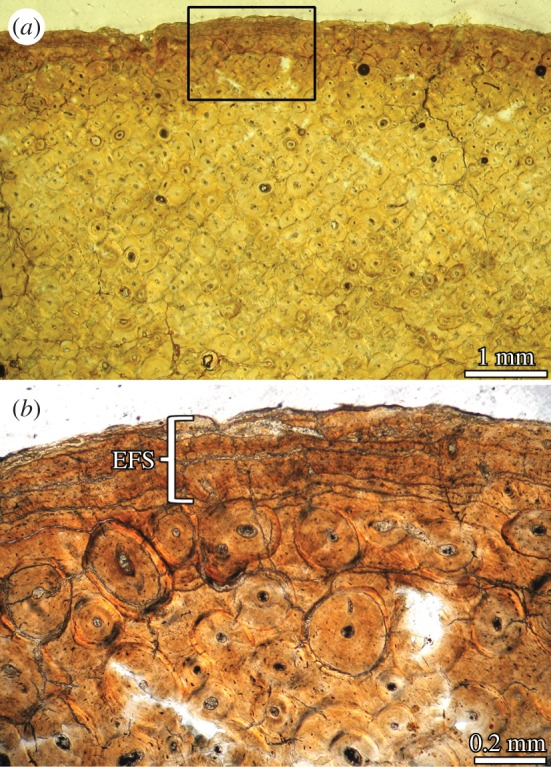
Femur histology of *Epachthosaurus sciuttoi* UNPSJB-PV 920. (*a*) General view of the mid-diaphyseal cortex in transverse section. Note the extensive remodelling throughout the cortex. (*b*) Detailed view of the outermost cortex (box inset in (*a*)), showing the presence of an avascular external fundamental system (EFS).

Besides the nature of the ossified soft tissue, an important question concerns the causes that provoke such ossification. A possible answer is related to a pathological condition. Pathological ossifications of different types of spinal ligaments are commonly reported in several vertebrate groups and are especially well documented in humans [55,58–61]; this commonly represents a process of physiologic ageing [59,62]. The hypothesis of an age-related pathological cause for the supraspinous ligament ossification in sauropod can be actually tested using morphological and histological data. In this sense, if the individuals that possess an ossified ligament exhibit juvenile–subadult features (e.g. unfused neurocentral sutures, absence of an external fundamental system (EFS or outer circumferential layer) in the outer cortex of the compact bone), then age-related pathology is not supported. Both *E. sciuttoi* and MDT-Pv 4 do not reveal unfused neurocentral sutures in their vertebral column ([9]; G. A. Casal 2015, personal observation). Also, histological examinations of the femur of *E. sciuttoi* reveal the presence of an EFS (figure 6). These observations indicate that these specimens were somatically adults [30]. Although evidence for a juvenile or subadult ontogenetic stage in individuals with an ossified supraspinous ligament allows rejecting the hypothesis of an age-related pathology, the opposite observation cannot be used to reject the non-pathological hypothesis (the structure could be still formed before the reaching of somatic maturity). It is interesting to note that in both *E. sciuttoi* and MDT-Pv 4 the degree of secondary remodelling in the ossified ligament is strong, which indicates that the ligament was mineralized long before the individual's death. Nevertheless, it is not possible to establish if the ligament was ossified after or before the onset of somatic maturity. Unfortunately, there is no histological data from other sauropods with an ossified supraspinous ligament (e.g. *Malawisaurus*). Future histological studies on these specimens probably will shed light on this issue.

Another line of evidence to test the pathological hypothesis came from the histology of the ossified ligament. Microstructural studies on pathologically ossified ligaments reveal that important histological modifications in the soft tissue occur before the mineralization. In this sense, the commonly regular arrangement of the elastic and collagenous fibres disappears, elastic fibres tend to break and disappear, collagenous fibres become hypertrophied and a matrix of fibrocartilage proliferates [55,59–62]. None of these histological features were recorded in the primary bone matrix of the ossified ligament in our sample, which suggests a non-pathological origin for this structure.

A non-pathological cause for the mineralization of the supraspinous ligament in sauropod dinosaurs could be related to tensile forces supported for this structure in the sacral region. In this regard, among many birds, tendinous structures have a marked tendency to ossify under the influence of tensile force and in the absence of deformation [38,63]. As the ossified ligament in the sacrum of *E. sciuttoi* and MDT-Pv 4 was possibly part of a large, continuous ligament that ran along both presacral and caudal vertebrae tips (see below), an important tensional force was applied to this structure at the level of the sacrum. Also, given that the sacral vertebrae are co-ossified, no important deformation occurs in this part of the vertebral column. The continuous tension of the ligament and the absence of deformation in the sacral region possibly induced the mineralization of the ligament in this region of the skeleton. The non-pathological mineralization possibly had a functional advantage, providing a strong region of attachment to the unossified anterior and posterior segments of the complete supraspinous ligament.

### 4.3 Implications for soft tissue reconstruction

Whether the ossification of the supraspinous ligament was pathological or non-pathological, its identification in sauropod dinosaurs is important because it provides new information about the soft tissue anatomy of this group of vertebrates. The presence of a supraspinous ligament in sauropod dinosaurs was early suggested by Janensch [64,65], who proposed that a single supraspinous ligament extended from the anterior-most, non-bifurcated dorsal vertebra to the anterior caudal vertebrae in sauropods with bifurcated vertebrae, such as *Dicraeosaurus hansemani* [64]. Also, he proposed that a long ligament extended from the cervical to the caudal vertebrae in sauropods that lacked bifurcated vertebrae, such as *Girafatitan brancai* [65]. The ossified ligament in *E. sciuttoi* and other sauropods could be part of this long supraspinous ligament. Taylor & Wedel [66] showed that the presacral vertebrae of some sauropod taxa (e.g. *Sauroposeidon proteles*, *Mamenchisaurus hochuanensis*) possess rugose neurapophyses with spurs directed anteriorly and posteriorly from the tip of the spine. They proposed that these structures either anchored discontinuous interspinous ligaments or were embedded in a continuous supraspinous ligament. If the latter condition is correct, such a ligament could be continuous with the supraspinous ligament that ossifies at the level of the sacrum. As reported in other sauropods (e.g. Titanosauridae indet. MCT 1489-R), the ossified supraspinous ligament of *E. sciuttoi* becomes wider toward the anterior portion [9,12,16]. This variation could be related to a major development of the supraspinous ligament in the presacral vertebrae. In this sense, and as occurs in mammals [67], the supraspinous ligament could be fused with the tendon insertions of the *longissimus dorsi* muscle in the dorsal region.

The caudal extension of the supraspinous ligament probably reaches the posterior region of the tail. The presence of an important supraspinous ligament in the tail of the sauropod and other dinosaurs has been previously mentioned [68] and is supported by the striated appearance of the dorsal surface of the neural arches (e.g. [69]) and, in some cases, by the partial ossification of this ligament in some specimens.

In conclusion, our data support the hypothesis of a supraspinous ligament in sauropod dinosaurs, which became ossified in the sacral region of some taxa. Although it is not possible to establish the total extension of the ligament along the vertebral column, it is probable that this structure developed along most of the presacral and caudal vertebrae. According to the data provided by Powell [15,16] and Campos & Kellner [12], the supraspinous ossified ligament described in an unnamed titanosaur from Brazil (MCT 1489-R) is actually dorsally divided by a ‘longitudinal ligamentary groove’. Interestingly, the bovine nuchal ligament is dorsally divided by a median sulcus filled with fatty elastic areolar tissue [50]. This dorsal division has not been reported in other ossified supraspinous ligaments in sauropods, including *E. sciuttoi* and MDT-Pv 4, which could be related to interespecific variations.

### 4.4 Ossified supraspinous ligament in sauropods

**Figure 7.**
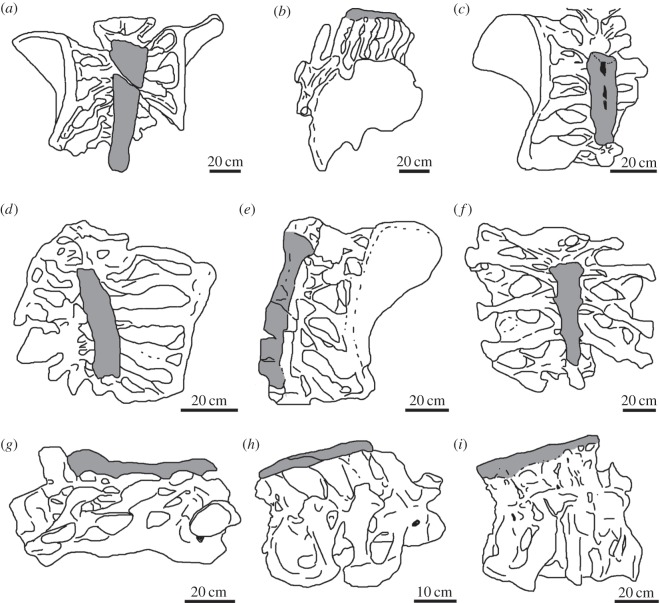
Supraspinous rod (dark grey) in the sacrum of *Epachthosaurus sciuttoi* (*a*); Lithostrotia indet. MDT-Pv 4 (*b*); Titanosauridae indet. MCT 1489-R (*c*); *Malawisaurus dixeyi* (*d*); *Huanghetitan liujiaxiaensis*(*f*); *Huanghetitan ruyangesis* (*g*); *Atsinganosaurus velauciensis (h)*; and *Tastavinsaurus sanzi* (*i*). (*a*,*c*–*f*) dorsal view; (*b*) left lateral view; (*g*–*i*) right lateral view. Drawings based on: Casal *et al*. [11] (*b*); Campos & Kellner [12] (*c*); Gomani [10] (*d*); D'Emic *et al*. [71] (*e*); You *et al*. [70] (*f*); Lü *et al*. [72] (*g*); García *et al*. [13] (*h*) and Canudo *et al*. [73] (*i*).

Because some authors [9,10,13,70] have considered that the ossification of the supraspinous ligament at the level of the sacrum is a character of systematic value, here we discuss its distribution among sauropod dinosaurs in a phylogenetic context. The ossified ligament in the sacrum has been described in a total of 10 specimens from nine taxa (figure 7), including: *E. sciuttoi* [9,17,74], *Atsinganosaurus velauciensis* [13], *M. dixeyi* [10], Titanosauridae indet. MCT 1489-R [12,16], *Huabeisaurus allocotus* [71], *Huanghetitan ruyangesis* [72], *Huanghetitan liujianxensis* [70], *Tastavinsaurus sanzi* [73] and Lithostrotia indet. MDT-Pv 4 ([11], this work). Although García *et al*. [13] mention that the ossified ligament is also present in the Brazilian titanosaur *Baurutitan britoi*, there is no report of this structure in the original descriptions of this taxon [15,75].

**Figure 8.**
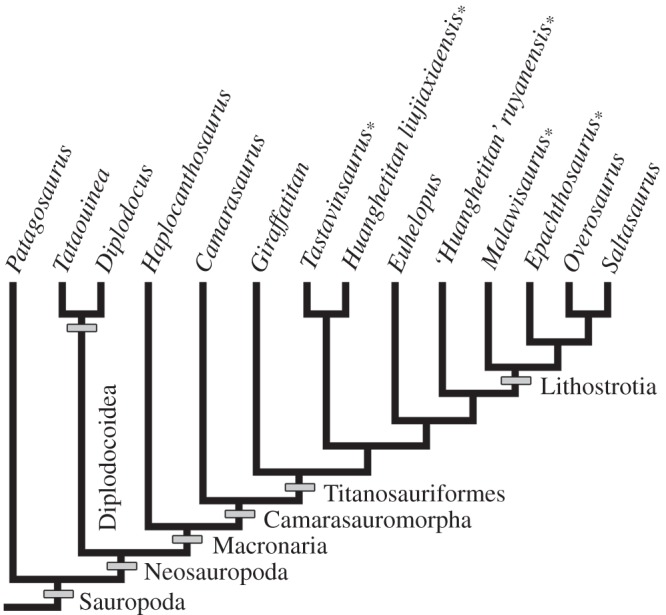
Phylogenetic relationships of different sauropod taxa for well-preserved sacra are reported. Asterisks indicate the presence of supraspinous ossified ligament in the sacrum. Tree topology is based on Carballido & Sander [76], Coria *et al*. [77], Fanti *et al*. [78], Mannion *et al*. [79] and Salgado *et al*. [80].

The phylogenetic distribution of the ossified supraspinous ligament in sauropod dinosaurs is difficult to assess given that (i) not all the mentioned taxa have been included in published cladistic analysis (e.g. Titanosauridae indet. MCT 1489-R, *A. velauciensis*); (ii) no more than three of these taxa have been included in a single analysis; and (iii) the phylogenetic position of some taxa varies between analyses (e.g. *T. sanzi*). Despite these difficulties, some general trends are observed (figure 8). In all the phylogenetic analyses, at least four taxa (*E. sciuttoi*, *M. dixeyi*, *H. ruyangesis* and*H. liujianxensis*) are always recovered as members of Titanosauriformes (e.g. [26,76,79–83]). In the case of *T. sanzi*, this European sauropod has been considered either as a member of Titanosauriformes [26,73,79,80,83] or a basal Camarasauromorpha [76,82]. In the case of those taxa not included in published phylogenetic analysis (*A. velauciensis*, *H. allocotus* and Titanosauridae indet. MCT 1489-R), synapomorphic characters allow their assignment in different positions within Titanosauriformes [12,13,16,71]. Finally, as previously mentioned, specimen MDT-Pv 4 can be included within Lithostrotia. These data reveal that the presence of a sacral ossified ligament is restricted to Titanosauriformes (or Camarasauromorpha following the phylogenetic hypothesis of Carballido and colleagues [76,82]). As several well-preserved sacra in non-titanosauriform taxa (e.g. *Patagosaurus fariasi*, *Diplodocus longus*, *Camarasaurus lewisi*, *Haplocanthosaurus priscus*) lack an ossified supraspinous ligament, its absence do not appears to be a preservation artefact [84–87]. Although the character appears to be present only in Titanosauriformes, it is clearly absent in several members of this clade (e.g. *Overosaurus paradosorum*, *Euhelopus zdanskyi*), particularly in derived lithostrotians [77,88]. At this point, the question is: if, as previously discussed, the ossification of the supraspinous ligament is not a pathological condition, why is the ossified ligament only present in Titanosauriformes (or Camarasauromorpha), but not in all of them? Possibly the capacity to ossify the supraspinous ligament is a homoplastic character that evolved early in Titanosaurifomes, but was lost in different taxa. It will be interesting and useful if future phylogenetic studies include all the taxa with sacral ossified supraspinous ligament in the same analysis and, moreover, if this feature is included as a morphological character.

## 5. Conclusion

Bone histology indicates the presence of an ossified supraspinous ligament in the sacrum of some sauropod taxa. The origin of this structure appears to be related to a non-pathological metaplastic ossification, possibly induced by the continuous tensile forces applied to the element. The ossified ligament is perhaps related to a larger structure that extended toward the presacral and caudal vertebrae, which only was ossified at the sacral region. Although previous authors have proposed that the ossified ligament served as a reinforcement for the sacrum [9,16,17,89], given that this region of the column is actually co-ossified in sauropods, we judge that hypothesis as improbable. In this regard, we consider that the ossification of the supraspinous ligament at the sacrum level possibly provided a strong attachment site for the non-ossified portions (presacral and caudal) of the same ligament.
